# Integrons in the development of antimicrobial resistance: critical review and perspectives

**DOI:** 10.3389/fmicb.2023.1231938

**Published:** 2023-08-31

**Authors:** Basharat Ahmad Bhat, Rakeeb Ahmad Mir, Hafsa Qadri, Rohan Dhiman, Abdullah Almilaibary, Mustfa Alkhanani, Manzoor Ahmad Mir

**Affiliations:** ^1^Department of Bio-Resources, School of Biological Sciences, University of Kashmir, Srinagar, India; ^2^Department of Biotechnology, School of Life Sciences, Central University of Kashmir, Ganderbal, India; ^3^Department of Life Sciences, National Institute of Technology (NIT), Rourkela, Odisha, India; ^4^Department of Family and Community Medicine, Faculty of Medicine, Al Baha University, Al Bahah, Saudi Arabia; ^5^Department of Biology, College of Science, Hafr Al Batin University of Hafr Al-Batin, Hafar Al Batin, Saudi Arabia

**Keywords:** antimicrobial resistance, antibiotic stewardship, horizontal gene transfer, integrons, pathogenicity

## Abstract

Antibiotic resistance development and pathogen cross-dissemination are both considered essential risks to human health on a worldwide scale. Antimicrobial resistance genes (AMRs) are acquired, expressed, disseminated, and traded mainly through integrons, the key players capable of transferring genes from bacterial chromosomes to plasmids and their integration by integrase to the target pathogenic host. Moreover, integrons play a central role in disseminating and assembling genes connected with antibiotic resistance in pathogenic and commensal bacterial species. They exhibit a large and concealed diversity in the natural environment, raising concerns about their potential for comprehensive application in bacterial adaptation. They should be viewed as a dangerous pool of resistance determinants from the “One Health approach.” Among the three documented classes of integrons reported viz., class-1, 2, and 3, class 1 has been found frequently associated with AMRs in humans and is a critical genetic element to serve as a target for therapeutics to AMRs through gene silencing or combinatorial therapies. The direct method of screening gene cassettes linked to pathogenesis and resistance harbored by integrons is a novel way to assess human health. In the last decade, they have witnessed surveying the integron-associated gene cassettes associated with increased drug tolerance and rising pathogenicity of human pathogenic microbes. Consequently, we aimed to unravel the structure and functions of integrons and their integration mechanism by understanding horizontal gene transfer from one trophic group to another. Many updates for the gene cassettes harbored by integrons related to resistance and pathogenicity are extensively explored. Additionally, an updated account of the assessment of AMRs and prevailing antibiotic resistance by integrons in humans is grossly detailed—lastly, the estimation of AMR dissemination by employing integrons as potential biomarkers are also highlighted. The current review on integrons will pave the way to clinical understanding for devising a roadmap solution to AMR and pathogenicity.

**Graphical Abstract:**
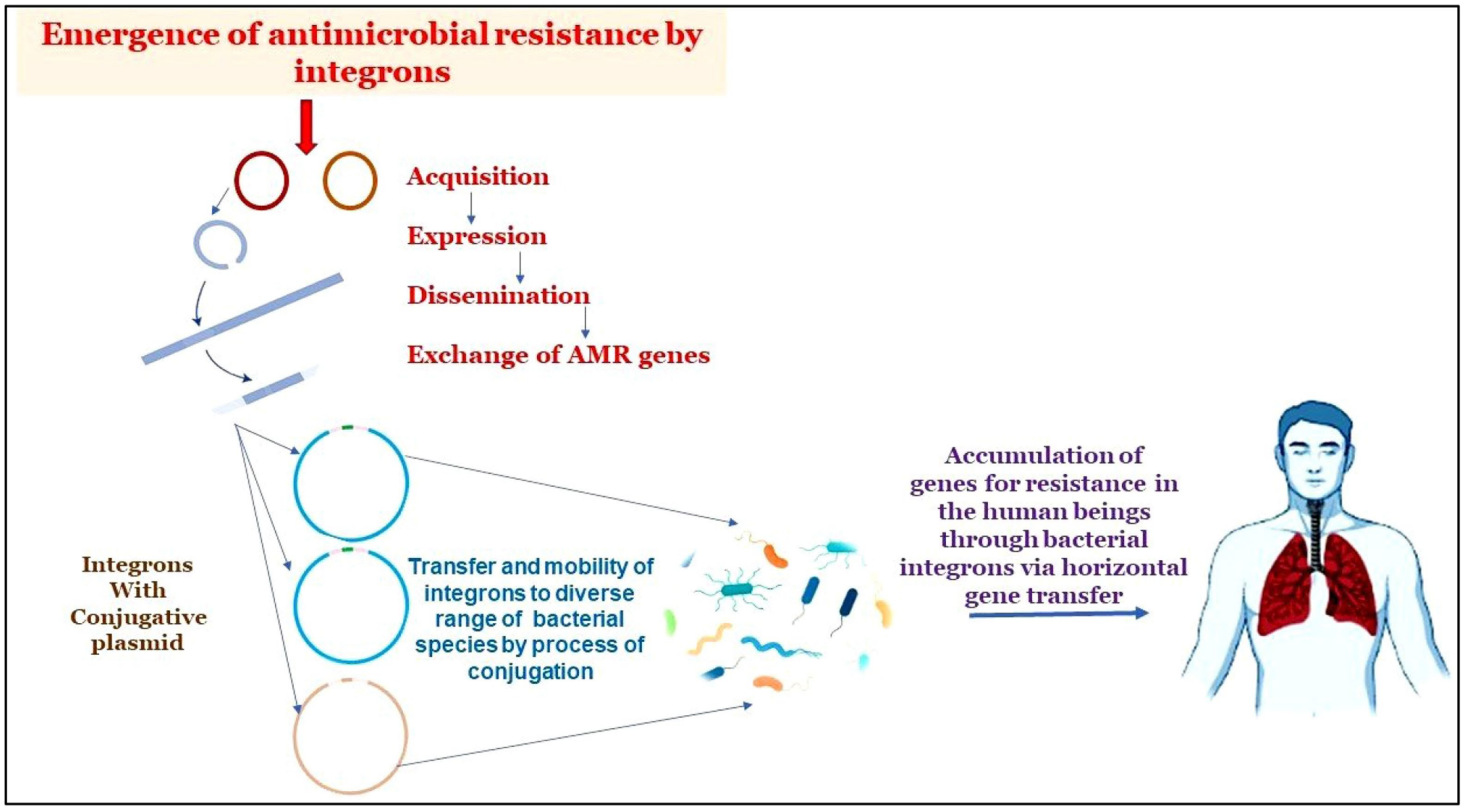
The graphical abstract displays how integron-aided AMRs to humans: Transposons capture integron gene cassettes to yield high mobility integrons that target res sites of plasmids. These plasmids, in turn, promote the mobility of acquired integrons into diverse bacterial species. The acquisitions of resistant genes are transferred to humans through horizontal gene transfer.

The graphical abstract displays how integron-aided AMRs to humans: Transposons capture integron gene cassettes to yield high mobility integrons that target res sites of plasmids. These plasmids, in turn, promote the mobility of acquired integrons into diverse bacterial species. The acquisitions of resistant genes are transferred to humans through horizontal gene transfer.

## Highlights

- We highlighted the detailed molecular mechanism mediated by integrons to serve as natural cloning and expression vectors to disseminate antimicrobial resistance genes in various microbial species.
- The review emphasizes highlighting integrons as genetic markers for estimating AMR existing in microbial species and their existence in humans.
- The current study underlines the worldwide risk to human health posed by integron-mediated antibiotic-resistance gene cassettes and their transmission to humans.
- The review highlights the spread of AMR through integrons as a global issue. Additionally, we raised concerns about devising strategies to hinder the resistance mechanisms evolved by microbes through the aid of integrons.

## Introduction

The mounting threat posed by antimicrobial resistance and engraved pathogenicity on humans forced scientific communities to profoundly investigate the evolution of resistance mechanisms and their responsible agents (Hamdani et al., 2020; Larsson and Flach, 2022; Sheikh et al., 2022). Antibiotic abuse and overuse have resulted in the formation and dissemination of antibiotic-resistance genes (ARGs) and antibiotic-resistant bacteria (ARBs), causing widespread worry around the world in human medicine and livestock breeding (Serwecińska, 2020; Mir et al., 2021; Sheikh et al., 2021b). Since ARGs are commonly found in domestic sewage, mud/dirt, and animal waste in these environments, these ecosystems are considered key reservoirs for ARGs (Wellington et al., 2013; Ding et al., 2019) to many bacterial species, particularly to those causing disease. The ultimate, well-known effects of such accumulated evolutionary events are gradually growing challenges in preventing and treating bacterial diseases. Understanding and recognizing human, animal, and environmental microbiota relationships is crucial because bacteria and genes frequently cross habitats and species boundaries “One Health Concept” (Collignon, 2012; Zinsstag et al., 2012; So et al., 2015; Torren-Edo et al., 2015; World Health Organization, 2015; Collignon and McEwen, 2019) to manage this global health challenge (Wellington et al., 2013; Berendonk et al., 2015; Bengtsson-Palme et al., 2018). Antimicrobial resistance is an ecological issue that affects the wellbeing of people, animals, and the environment. It is characterized by complex interactions involving many microbial populations (Fletcher, 2015; Collignon and McEwen, 2019).

The establishment and prevalence of antimicrobial resistance are caused by various resistance mechanisms, which may be divided into genetic mutation occurring at a low frequency and acquisition of different genes mediated resistance to their host microbes. As a result, it has been determined that acquiring resistance genes is a significant factor in antibiotic resistance’s widespread distribution and spread. The resistance is accomplished through vertical or horizontal transfer, with the latter method involving mobile genetic elements like plasmids and transposons (Xu et al., 2011b). Integrons are primarily carried by plasmids or contained within transposons, and their mechanism and function in the movement of microorganisms are well-known and documented (Hall and Collis, 1995; Mazel, 2006), which had also been considered to contribute to the unleashing of “Super Bugs” (Xu et al., 2011b,c).

The exchange of genetic material between bacteria of the same generation is known as horizontal gene transfer (HGT). The heredity of the transferred sequences in the recipient microbe is also crucial for a successful HGT event, in addition to the entry of DNA into the cytoplasm of the recipient cell (Ochman et al., 2000). HGT influences bacterial population’s genetic diversity and evolutionary trajectories. For instance, the main factor causing the establishment, recombination, and spread of multidrug resistance among bacterial pathogens is the HGT of mobile genetic elements (MGEs) (Nakamura et al., 2004; Thomas and Nielsen, 2005). MGEs are widespread in bacterial populations due to their ability to physically migrate between host genomes. MGEs include plasmids, bacteriophages, genomic islands (GIs), transposons (Tns), integrons, insertion sequences (ISs), integrative and conjugative elements (ICEs), and tiny inverted-repeat transposable elements, among others (MITEs) (Stokes and Gillings, 2011).

Mobile genetic elements (GEs), such as integrons, may be critical in transferring ARGs across microbial organisms in their surroundings. Integrons may extract ARGs from their surroundings and subsequently incorporate them into their gene cassettes via location-specific recombination (Zhang et al., 2009; Sheikh et al., 2021a). Integrons are thus crucial in developing antibiotic tolerance and the horizontal gene transfer (HGT) of ARGs between bacterial organisms in diverse settings. The discovery of mobile integrons as critical players in the acquisition, mobilization, shuffle, and initiation of expression of gene cassettes, especially those encoding antibiotic resistance (Domingues et al., 2012; Escudero et al., 2015). The genetic elements known as integrons have a location-specific recombination system that enables them to acquire, express, and transfer particular DNA fragments known as gene cassettes (Hall and Collis, 1995).

Integrons are genetic elements that contain a site-specific recombination system able to integrate, express and exchange specific DNA elements, called gene cassettes (Hall and Collis, 1995). The integron is not regarded as a mobile element because it lacks mobility-related functions. In contrast, the gene cassettes contained in integrons are regarded as mobile, even though the spontaneous exchange of gene cassettes is rarely observed experimentally (Guerin et al., 2009; Baharoglu et al., 2010). Nevertheless, sequence-similar integrons appear widespread among bacterial species and genetic backgrounds, suggesting that they are frequently exposed to mechanisms that allow them to disseminate horizontally through bacterial populations (Stokes and Hall, 1989).

The present review article aimed to update the account of gene cassettes harbored by integrons on resistance and pathogenicity and searched to assess existing AMRs and antibiotic resistance by integrons in humans. Lastly, estimating AMR dissemination by employing integrons as potential biomarkers is also highlighted to have an in-depth role in global AMR dissemination. We believe this review will help to understand better how to develop a plan to minimize the global phenomenon of increasing antimicrobial resistance and pathogenicity related to human pathogenic microbes.

### Integrons

An integron is typically characterized by the presence of an integrase gene (*intI*) located near a primary recombination site known as attI (Deng et al., 2015). Structurally, all integrons consist of three main components, including 5′ and 3′ conserved segment and a central variable region between the 5′ and 3′ zone, in which integrons are responsible for the capture and expression of exogenous genes, which are part of the gene cassette. Integrons are accountable for capturing and expressing the exogenous genes that form the gene cassette in this central variable region (Mazel, 2006). The first component of the integron is a gene which produces a tyrosine recombinase (integrase, encoded by the intI gene), required for site-specific recombination inside the integron. The second component is an adjacent recombination site (attI), which the integrase recognizes. The third component is a promoter (Pc), which is situated upstream of the integration and required for the effective process of transcription and expression (Mazel, 2006; Labbate et al., 2009). The promoter aids in the expression of any integrated gene(s). The tyrosine-specific recombinase family, which also comprises the well-known integrase of the λ-phage, includes integrase (IntI) (Grainge and Jayaram, 1999; Carattoli, 2001).

**Figure 1:**
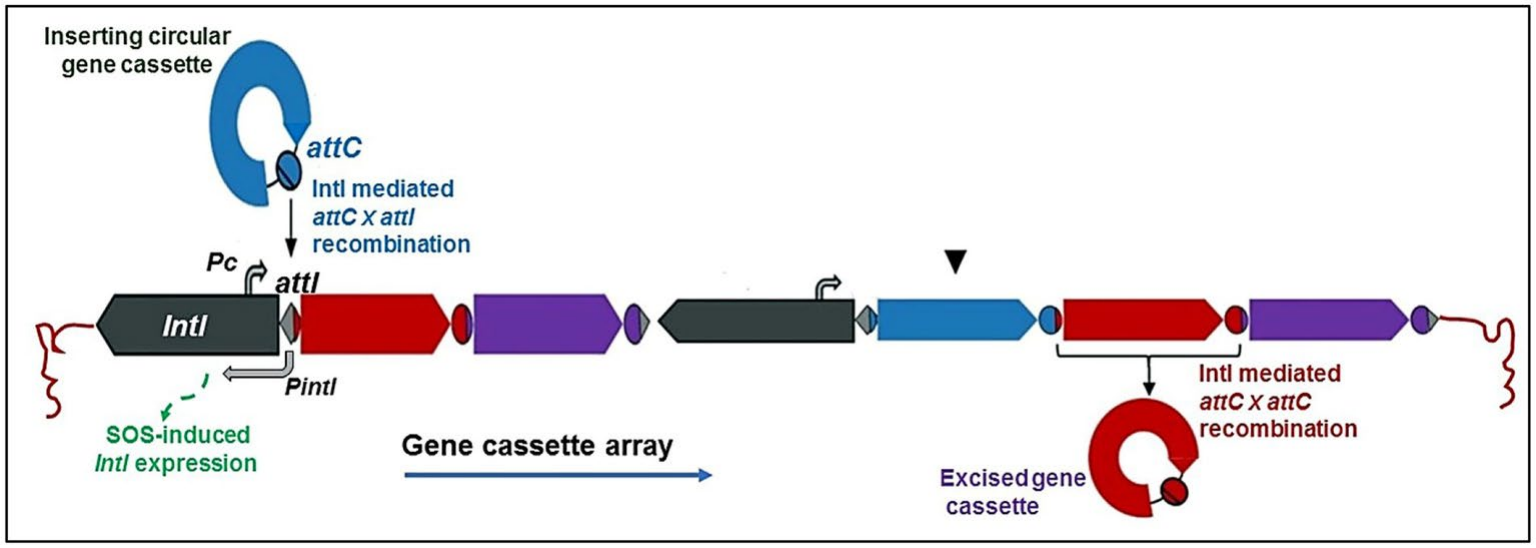
A schematic diagram of a class 1 integron displays its essential components: the P1 promoter, which drives gene cassette transcription; the P2 promoter, typically inactive; the int gene encoding the integrase enzyme; the attI1 integration site; the qacE11 gene conferring resistance to quaternary ammonium compounds; the sul1 gene for sulfonamide resistance; an orf5 of unknown function; and associated P promoters regulating expression of resistance genes. The attC site on gene cassettes is specifically recognized by the integrase for recombination (Deng et al., 2015).

These three components are simple structures with a single open reading frame (ORF) surrounded by a cassette-associated recombination site. They were initially called 59-base elements but are now referred to as *attC* (Gillings, 2014). While the Pc promoter is believed to be present and active in all forms of integrons, this has not been demonstrated for all of them (Ghaly et al., 2021a). Numerous integrase genes found in integrons have LexA binding sites close to their promoter regions allowing them to be regulated by the host LexA protein, which functions as a transcriptional repressor of the SOS response; as demonstrated for a class 1 integrase and in an integrase present in a CI (chromosomal integron) of *Vibrio cholerae* (Guerin et al., 2009; Baharoglu et al., 2010). These findings show that SOS induction can result in increased integrase gene transcription and activity and cassette rearrangements in organisms possessing lexA alleles (Baharoglu et al., 2010) The modulation of integrase transcription is unknown without a LexA ortholog. Integrons might have a single gene cassette or many of them (Hall and Collis, 1995). In most clinical bacterial strains, integrons have no more than 5 cassettes (Bennett, 2008), but integrons containing at least 9 genes for antibiotic tolerance have also been found. (Naas et al., 2001). The INTEGRAL database^1^ containing a list of numbered C1 integrons is available (Moura et al., 2009).

Initially, integrons were grouped into a few categories based on their genetic similarity to the integrase intI gene sequence, with classes 1, 2 and 3 receiving major attention (Nield et al., 2001; Domingues et al., 2012). Nevertheless, more DNA sequencing-based research have revealed more of the integrase gene’s genetic diversity and discovered over 90 distinct gene variations, questioning the initial classification system in doubt (Gillings et al., 2008; Domingues et al., 2012). Class 4 integron was discovered later in a relationship with chromosomal integrons. *IntI* encodes an integrase (IntI) of the tyrosine recombinase family, which is distinguished by the presence of invariant RHRY (with Y being the catalytic tyrosine) amino-acids in the conserved motifs named box 1 and box 2 (which distinguish intI from other XerC-related integrases) (Xu et al., 2011a). IntI-catalyzed recombination between attI and/or attC sites results in the insertion or excision of cassettes (Figure 1). The class 1 integrase (IntI1) recognizes three types of recombination site: attI1, attC and secondary sites. Binding domains and consensus sequences have been determined for these. The attI1 site is a simple site which contains two inverted sequences that bind the integrase and two additional integrase-binding sites known as strong (DR1) and weak (DR2) (Francia et al., 1999; Hall et al., 1999).

Further, Integrons are categorized into 3 primary categories based on their phylogenetic analyzes of sequenced intI genes: the marine γ-proteobacteria group, the soil/freshwater proteobacteria group, and the inverted integrase group (Domingues et al., 2012). C1 integrons are the most common and pervasive, especially in clinical environments, and significantly contribute to the development of antibiotic tolerance (Amin et al., 2021). Although the origin of C1 integron is unknown, it is hypothesized to have existed in different bacterial species before the “antibiotic age (Stokes et al., 2006).” C1 integrons are present in the environment without antibiotic-resistant genes. The initial source of these genomic structures has been speculated to be beta proteobacteria. Tn*402* (also known as Tn*5090*) characteristic features are absent in such types of environmental integrons suggesting that they were integrated into a plasmid-borne Tn*402* transposon carrying a gene cassette imparting host advantage (Gillings et al., 2008). For instance, the *qac* gene, which imparts resistance to quaternary ammonium compounds and thus biocides, has a long history of use in clinical usage; and the su1I gene, confers resistance to sulphonamides. The early association of the Tn*402* transposon’s progenitor with a class 1 integrase and an attI1 site is another possible origin of the C1 integrons. Most C1 integrons have the 5’-CS region in the same location (Toleman and Walsh, 2011). The 3’-CS region is thought to be the result of a fusion of the Tn402 transposon’s *qacE* gene with the su1I gene, followed by partial deletion of the *qacE* gene; this fusion occurred simultaneously with a deletion event in the Tn*402* transposon’s transposition functions, resulting in a structure incapable of self-mobilization (Toleman and Walsh, 2011). Although a few C1 integrons with a complete transposition module have been found, the bulk of C1 integrons are transposons with incomplete transposition modules (Juan et al., 2010; Marchiaro et al., 2010). After the initial generation of C1 integrons, intense selection for antimicrobial tolerance favored the detection of antibiotic tolerance gene cassettes giving rise to the C1 integron components that are familiar to us today (Bennett, 2008; Stokes and Gillings, 2011).

Integrons are further categorized according to their environment, with two distinct types: mobile integrons (MIs) and chromosomal integrons (CIs) (Liebert et al., 1999). CIs are composed of a variable number of gene cassettes between zero and hundreds, the majority of which do not contribute to antimicrobial drug tolerance. Although migration of gene cassettes from CIs has been recorded, CIs are considered sedentary (Liebert et al., 1999; Domingues et al., 2012). In comparison, MIs like C1 integrons carry a restricted number of gene cassettes and are frequently engaged in antimicrobial tolerance propagation (Domingues et al., 2012).

### attC SITES

The *attC* region consists of two symmetrical sites, each comprising conserved short core sequences (7–8 base pairs) designated as Rʹ and Rʹʹ, and Lʹ and Lʹʹ. The Rʹ and Rʹʹ sequences align with the RH consensus, while Lʹ and Lʹʹ correspond to the LH consensus. These sequences are believed to guide the integrase in recognizing orientation-specific integration. The Lʹʹ site is especially important for determining insertion direction, and the LH site enhances the recombination efficiency of the RH site. Typically, an *attC* site is linked to a single open reading frame (ORF), forming a gene cassette. These cassettes, although sometimes independent, become a functional part of the integron upon integration (Deng et al., 2015).

### Gene cassettes

Studies have shown that the variable region of integrons may occasionally lack gene cassettes. When present, cassettes are integrated into the integron structure via site-specific recombination between *attI* and *attC* sites. These gene cassettes may exist as unstable circular DNA elements or as linear forms following directional insertion. Although they possess coding sequences, gene cassettes typically lack promoters and rely on the integrons promoter for expression. More than 130 distinct antibiotic resistance genes have been identified within these cassettes, conferring resistance to a broad range of antimicrobial agents, including b-lactams, aminoglycosides, chloramphenicol, streptothricin, trimethoprim, rifampin, erythromycin, quinolones, fosfomycin, lincomycin, and quaternary ammonium compounds.

Antimicrobial resistance arises through both rare spontaneous mutations and, more significantly, acquisition of resistance genes via vertical and horizontal gene transfer. The latter is often mediated by mobile genetic elements such as plasmids and transposons. Integrons, frequently located on these elements, facilitate rapid bacterial adaptation and are implicated in the rise of multidrug-resistant organisms, often referred to as “superbugs” (Deng et al., 2015).

### Site-specific insertion of gene cassettes into the integron

The integration of circular gene cassettes occurs by site-specific recombination between *attI* and *attC*, assisted by the integron integrase. The first resident gene cassette’s 5′ end should be the target of insertion. The attI site in the 5’-CS conserved segment of the integron and the *attC* site in the 3’-CS conserved segment of the integron are the two key recombination sites where the integrase interacts with each gene cassette. For four sites inside the attI site, IntI has a high binding affinity (Partridge et al., 2018). It also weakly binds to the cassette’s *attC* site (Holmes et al., 2003). This procedure is reversible, and cassettes can be removed as free circular DNA elements (Johansson, 2007). Insertion at the attI site enables the expression of an incoming cassette driven by the neighboring Pc promoter (Razavi et al., 2017). As a tool for genomic innovation, the integron system offers two significant benefits. The bacterial genome initially incorporates additional genetic material through a specialized recombination site (attI) that inhibits the breakdown of existing genes. Secondly, the integron promoter (Pc), via which the freshly integrated gene is expressed, triggers the beginning of natural selection. Any newly generated variants will consequently instantly activate genes resulting in beneficial phenotypes in a population of cells with integrons, each with a unique gene cassette (Michael et al., 2004).An additional site in front of a gene not formerly present on cassettes may result in site-specific recombination between the attI and this secondary site, trapping the gene. The acquisition of the *attC* site, which is most likely achieved by a second stage of recombination from an existing gene cassette, is required to create a full cassette containing the new gene. Almost any gene may become an integron, although secondary-site recombination is uncommon and less effective than primary-site recombination. Integrons are further classified into two classes based on the genomic context of those genes: chromosomal integrons, which are found inside the bacterial chromosome, and mobile integrons, which are associated to transposons (Antelo et al., 2021; Sandoval-Quintana et al., 2022).

### Mobility of integron: spreading resistance genes on a global scale

**Figure 2:**
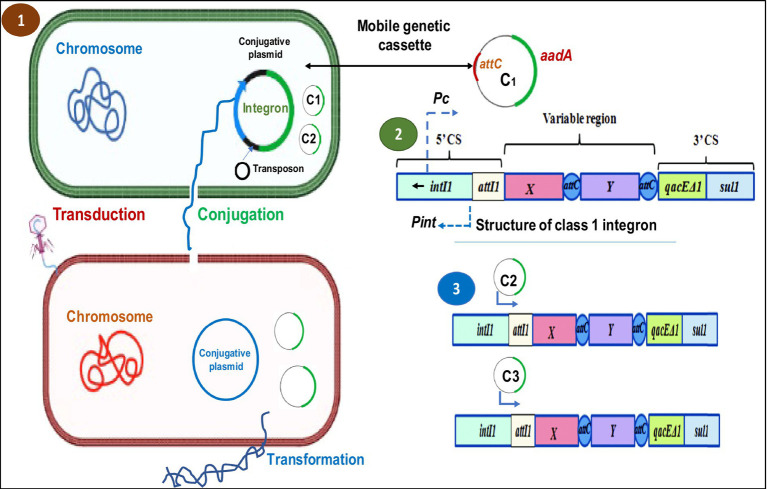
Schematic representation of HGT mechanisms in bacteria and the general structure of an integron and gene cassette. (1) HGT mechanism (conjugation, transduction and transformation) in bacteria. (2) The genetic organization of the class 1 integron DNA sequence includes the integrase gene (IntI) and one cassette gene. While the cassette array is expressed from the PC promoter, the integrase is expressed from the Pint promoter. (3) The orientation of insertion of the following genomic cassettes (C2, C3) into the integron structure. It is believed that as a gene is moved further away from the PC promoter, its expression level within the cassettes will decrease (Racewicz et al., 2020).

Integrons have been reported to be widespread and found across clinical species, and their movement has been cited as a primary source of concern for clinical antibiotic resistance. Integrons are connected with mobile DNA elements (transposons or plasmids) and antibiotic-tolerance genes, despite their small array dimensions and considerable heterogeneity in the sequence of *attC* sites (Verraes et al., 2013; Partridge et al., 2018). Although self-transposition is defective, existing integrons (mainly class 1 integrons) were thought to represent a potentially mobile genetic element. They’re typically seen on plasmids to aid conjugative transfer. They include mobile gene cassettes that can move to other integrons or secondary locations in the bacterial genome (Figure 2). Integrons may carry and propagate antibiotic tolerance genes, making them one of the most significant horizontal transfer pathways for transmitting tolerance/resistance genes across bacteria.

Integrons are a naturally occurring system of capturing and construction enabling microbes to incorporate gene cassettes and then correctly express them to transform them into functional proteins. Gene cassettes can be formed from various ORFs, and it is essential to understand how this process works. To allow for quick adaptability to selection pressure and hence boost the host’s overall fitness and advantage, integrons may be able to interchange and stockpile functioning gene cassettes forever (Yu et al., 2013; Deng et al., 2015). Frequent gene cassette arrays in various integron types and their impact on antimicrobial tolerance in various species of bacteria is depicted in Table 1.

**Table 1:** Frequent gene cassette arrays in various integron types and their impact on antimicrobial tolerance in distinct species of bacteria (Sabbagh et al., 2021).

Bacteria	Integrons	Gene cassettes	Antibiotics associated with gene cassettes	References
*Acinetobacter baumannii*	I, II	bla_CARB-2_, aadA1, aadA2, aadB, dfrA1, dfrA7, dfrA1-gcuF, dfrA1- aadA1, dfr17-aadA5, dfr12-gcuF-aadA2, sat1.	Extended Spectrum Beta–Lactamase, Aminoglycosides, Trimethoprim	Liu et al. (2014), Domingues et al. (2015), and Akrami et al. (2017)
*Klebsiella* spp.	I, II, III	bla_CARB-2_ bla_GES-1_, aadA, aadA1, aadB, dfrA1, dfrA7, dfrA1-gcuF, dfrA1- aadA1a, dfr17-aadA5, dfr12-gcuFaadA2.	Extended Spectrum Beta- Lactamase, Trimethoprim, Aminoglycosides,	Wu et al. (2013) and Domingues et al. (2015)
*Enterobacter* spp	I	aadA1a, aadA2, dfrA7, dfrA1-aadA1a, dfr17-aadA5, dfr12-gcuF-aadA	Aminoglycosides, Trimethoprim	Marathe et al. (2015)
*Enterococcus faecalis*	I	aadA1a, dfr12-gcuF-aadA2, dfrA1- sat1-aadA1.	Aminoglycosides, Trimethoprim	Sabbagh et al. (2017)
*Pseudomonas aeruginosa*	I	aadA2, aadB, dfr17-aadA5, dfr12-gcuF- aadA2.	Aminoglycosides, Trimethoprim	Ghaly et al. (2020a)
*Staphylococcus aureus*	I	aadA1, aadA2, dfr17-aadA5, dfr12- gcuF-aadA2, aacA4-cmlA1	Aminoglycosides, Trimethoprim, Chloramphenicol	Sow et al. (2010)
*Escherichia coli*	I, II, III	aadA1, aadA2, aadA5 aadB, dfrA1, dfrA5, dfrA7 dfrA12, dfr14, dfrA17, dfrB2, dfrA1-gcuC, dfrA1-aadA1, dfr17-aadA5, dfr12-gcuF-aadA2, dfrA1-sat1-aadA1, dfrA1-sat2-aadA1, estX-sat2-aadA1, blaOXA-101-aac (6′)–Ib, ere2.	Aminoglycosides, Trimethoprim, Extended Spectrum BetaLactamase, Erythromycin.	Kadlec and Schwarz (2008) and Kargar et al. (2014)
*Salmonella* spp.	I, II	aadA, aadA1a, aadA2, aadA5, aadB, dfrA1, dfrA7, dfrA12, dfrA17, dfrA1- gcuF, dfrA1-aadA1a, dfr17-aadA5, dfr12-gcuF-aadA2, bla_CARB-2_.	Aminoglycosides, Trimethoprim, Extended Spectrum BetaLactamase.	Antunes et al. (2006) and Domingues et al. (2015)

Furthermore, several reservoirs and genetic pools for integron, shared between bacteria, may be provided by mobile genetic elements, e.g., transposons, insertion sequences, etc. (Partridge et al., 2018). Integrons are essential for the spread and dispersion of genes associated with resistance. They transfer resistance genes to various drugs between bacteria (Deng et al., 2015). According to numerous findings on integrons from environmental microorganisms, including the high sequence diversity seen and the numerous functional products other than tolerance coded by these cassettes, integrons may have performed significantly in development and expansion for an extended period (Rendueles et al., 2018).

### Horizontal gene transfer and integrons

The primary mechanism by which antibiotic resistance spreads is horizontal genetic transfer (Sundström, 1998). Antibiotic resistance genes can also be transmitted via a variety of other mechanisms. One of the most significant forces influencing the development of microorganisms is horizontal gene transfer, or the acquiring of foreign deoxyribonucleic acid by microbes (Il’ina, 2003). It frequently causes the emergence of antibiotic tolerance (Huddleston, 2014; Ma et al., 2017). In clinical practice, most antimicrobial drugs come from or are derived from substances naturally found in the environment, primarily soil. Bacteria in the same domain as these compounds can pick up environmental genes for antibiotic tolerance. There is compelling evidence that this “environmental resistome” can be a rich source for acquiring antibiotic-tolerance genes in bacteria that are relevant to human health (Munita and Arias, 2016). This exchange of genetic material has been particularly linked to the spread of antibiotic tolerance to several widely used antibiotic drugs. The traditional methods by which bacteria acquire external genetic material are (i) transformation (the integration of bare DNA) (Venema, 1979; Johnston et al., 2014), (ii) transduction (mediated by phages) (Ozeki and Ikeda, 1968), and (iii) conjugation (Curtiss Iii, 1969). HGT, which involves transformation, is by far the most straightforward. However, only a few clinically significant bacterial species can “naturally” incorporate bare DNA to evolve antibiotic tolerance. Conjugation, a reliable method of DNA transfer that includes cell-to-cell contact and is expected to happen often in the human intestinal tract undergoing antibiotic therapy, is a prevalent reason for tolerance in the hospital setting. Although direct chromosome-to-chromosome transfer has been widely characterized, conjugation typically uses Mobile Genetic Components (MGEs) to convey important genetic information (Nwosu, 2001). Plasmids and transposons are the two most important MGEs because they substantially contribute to developing and expanding antibiotic resistance in therapeutically relevant organisms. The most effective methods for assembling antimicrobial tolerance genes are integrons, site-specific recombination systems that can attract open reading frames within mobile gene cassettes. Integrons offer a powerful and relatively straightforward method for adding novel genes to bacterial chromosomes and the tools needed to monitor their expression. They also promote beneficial genetic exchange and are a significant force in the development of bacteria (Munita and Arias, 2016).

**Figure 3:**
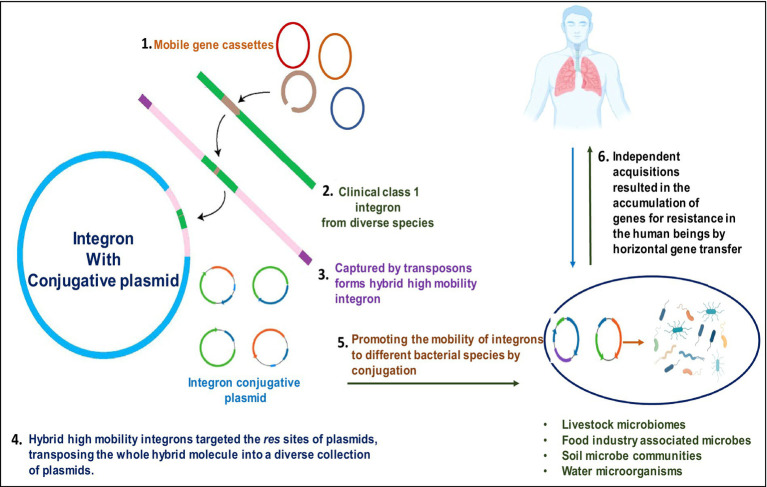
Pictorial representation of integron-aided AMRs to humans: (1) transposons capture integron gene cassettes to yield high mobility integrons that target res sites of plasmids. (2). These plasmids, in turn, promote the mobility of acquired integrons into diverse bacterial species. (3). The acquisitions of resistant genes are transferred to humans through horizontal gene transfer.

Integrons are a type of genetic component which allow exogenous genes to be efficiently captured and expressed and transfer the resistance genes through transposons and plasmids into bacterial species, which can inhabit humans (Figure 3). Their contribution to the antibiotic resistance expansion, particularly among bacteria, is well established. (Boucher et al., 2007). However, it is clear that integrons have a long evolutionary history dating back to their discovery in clinical settings and are a typical component of bacterial genomes. Integrons have been found in many habitats, can transfer between different species and lineages throughout evolutionary history, and can access a sizable number of new genes whose roles are primarily unknown. Integrons are much more significant than just being a characteristic of infections resistant to antibiotics, as evidenced by their function in bacterial adaptability and genome development in natural settings (Boucher et al., 2007).

### Human integron-antibiotic resistance gene cassettes: a rising threat

**Figure 4:**
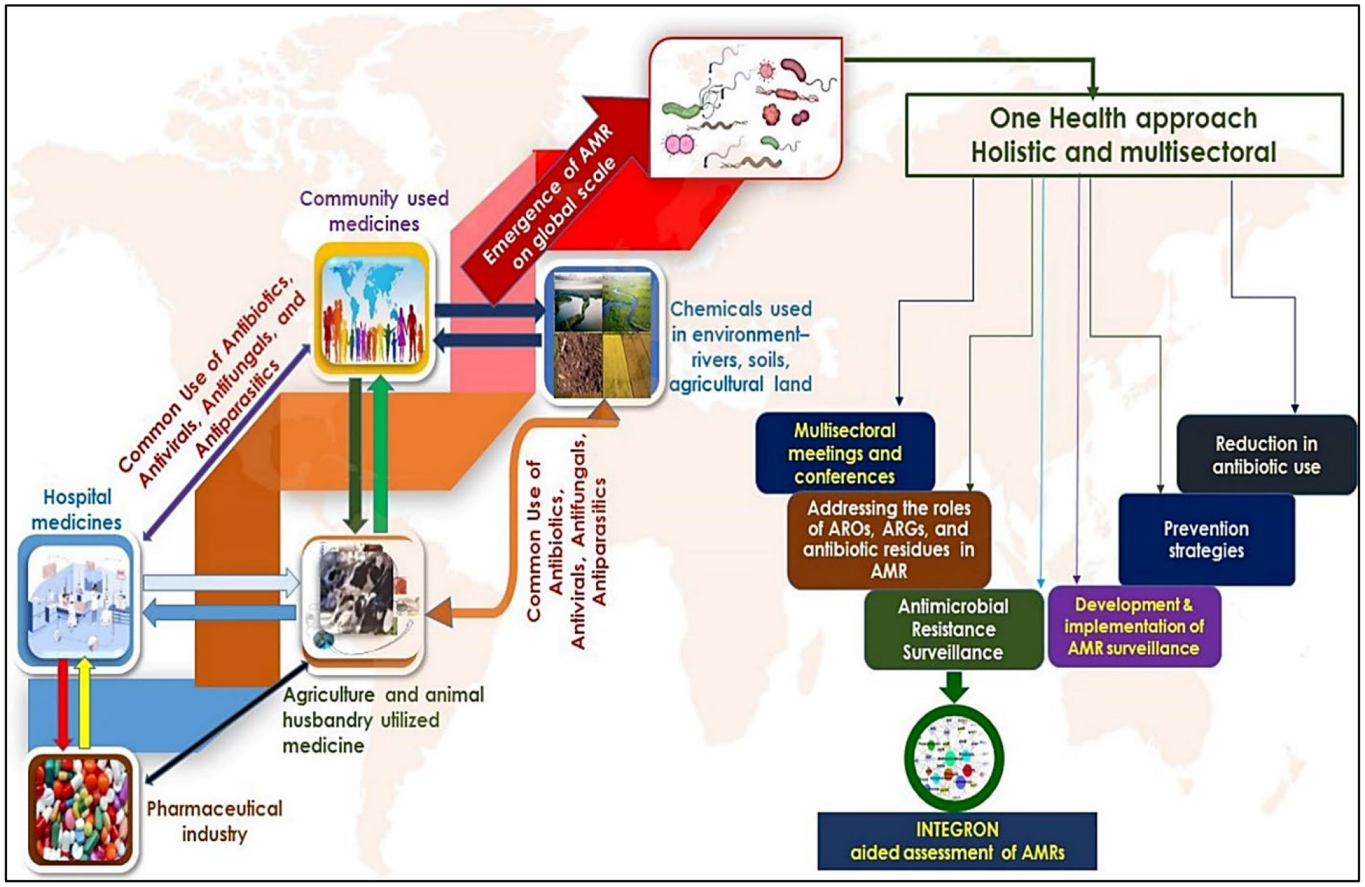
Dissemination and evolution of AMRs- the left side of the figure shows the routes through which antimicrobial resistance is developed and transmitted to different levels. Environmental habitats such as rivers, streams, and agricultural settings play a critical role in acquiring additional AMRs mainly disseminated by humans. The right panel of the figure shows the significance of the ‘One Health Approach’ in addressing the systematic assessment and clinical measures to control the AMRs, most especially by using integrons.

The resistance to antibiotics by microbe’s accounts for thousands of human deaths annually on a global scale and is continually increasing as a major threat to humans. Predictably, all the sections of researchers initiate the fight against antibiotic resistance, be it clinical settings, communities, or even agricultural systems, targeted to lower the prevention and transmission of antibiotic tolerance from one interface to another. To overcome this issue, several researchers have suggested initiating a “One-Health approach” to have a holistic viewpoint on antibiotic resistance at the level of animals, including humans, and dissemination patterns or routes in the environment (Collignon, 2013; So et al., 2015; Figure 4). Immense variability in genetic makeup, the higher opportunity for mutations, gene rearrangements, and horizontal gene transfer in microbes result in the higher establishment of microbial tolerance. These resistance genes are mobilized by mobile genetic factors, e.g., integrons. They are found in diverse habitats carried by various species of bacteria (Cury et al., 2016; Ghaly et al., 2019; Antelo et al., 2021; Dias et al., 2021). For instance, Dias et al. (2021) employed deep sequencing to explore the integron gene cassettes through studies of intI-*attC* amplicons. Integrons harboring antimicrobial resistance are critical in disseminating AMR genes through horizontal transfer via transposons and plasmids to develop multi-resistance (Roe et al., 2003; Gaze et al., 2013). The importance of tracing the resistome in colonizing the human gastrointestinal tract and cattle was investigated by the “One Health” approach, Chainier et al. (2017) by employing integrons as instrumental tools to assess the AMR carriage. The “One-Health approach” approach aims to promote the investigation of AMR dissemination through the integration of human health in conjunction with other animals (Chainier et al., 2017). Human feces samples are submitted to a high-throughput sequencing-based metagenomic analysis to study the prevalence and range of AMRs (Zhang et al., 2011; Ma et al., 2014, 2016).

Moreover, culture-dependent and culture-independent approaches are employed in checking the dissemination and surveillance of AMR (Chainier et al., 2017). Since integrons have remarkably evolved as potential markers to identify the AMRs in human digestive carriages, it is reported that integrons coding antibiotic resistance genes are copiously reported in commensal *E. coli* from human hosts on antibiotics doses (Kheiri and Akhtari, 2016).

Class 1 integrons are found most commonly out of all the different types of integrons. Several studies suggest that integrons are detected in various phyla such as *Actinobacteriota, Acidobacteriota, Cyanobacteria, Bacteroidota, Chloroflexota, Firmicutes, Campylobacterota, Chrysiogenetota, Desulfobacterota, Gemmatimonadota, Proteobacteria, Planctomycetota, Spirochaetota,* and *Verrucomicrobiota* (Cury et al., 2016; Parks et al., 2018; Table 2).

**Table 2:** Class 1 integron incidence and predominance of Gram-positive and Gram-negative microbial species (Deng et al., 2015).

Gram-negative bacterial strain	Class 1 Integron prevalence and the wide range of gene cassettes	Sampling	References
*Vibrio cholera*	44/176; *aadB-aadA2-blaP1-dfrA1-dfrA15*	Thailand	Dalsgaard et al. (2000)
*Pseudomonas aeruginosa*	45.8% (54/118)	Preliminary study in Guangzhou, China	Xu et al. (2009)
*Burkholderia*	29.4% (5/17); *oxa-aac* (6′-1a)	Ireland	Crowley et al. (2002)
*Campylobacter*	62/378	Ireland	Shin et al. (2015)
*Escherichia coli*	59.5% (355/597)	South Thailand	Vinué et al. (2008)
*Escherichia coli*		Preliminary study in Guangzhou, China	Xu et al. (2011b)
*Serratia*	1/30; *aacC1-ORFX-ORFY-aadA1*	Canada	Crowley et al. (2008)
*Salmonella*	36.2% (34/94); *aadA2-bla* (*PSE*-1) (61.76% 21/34); *aadA1-aadA2-bla* (*PSE*-1) (38.23%,13/34)	Animals, Japan	Ahmed et al. (2006)
*Stenotrophomonas maltophilia*	22% (20/93)	Kaohsiung Medical University	Chang et al. (2004)
*Salmonella enteritidis*	11.9% (59)	Taiwan	Huang et al. (2013)
*Aeromonas*	16/41 (39.02%); *dfrA15-cmlA4-aadA2*	Hidalgo, Mexico	Pérez-Valdespino et al. (2009)
*Enterobacteriaceae*	50/226	Addenbrooke’s Hospital	Machado et al. (2008)
*Escherichia coli*	4/32 (12.5%); *sat-1-aadA*	Meat and meat products, Norway	Sunde (2005)
*Klebsiella Pneumoniae*	18/26	Bloodstream infections	Xu et al. (2011a)
*Shigella*	*EstX-aadA1* (3.85%, 1/26)	Hiroshima prefecture, Japan; 2000–2004	Ahmed et al. (2006)
*Pseudomonas aeruginosa*	High prevalence	Iran	Shahcheraghi et al. (2010)

Class 1 integron is the most abundant and clinically significant. Class 1 integrons are crucial in disseminating AMR genes due to their higher mobility, distribution, and abundance than class 2 and 3 (Table 3; Gillings et al., 2008; Ghaly et al., 2021b). The present research on integrons has been on class 1 integrons, with gram-negative bacteria being the primary importance. The earliest multidrug resistance plasmids identified in the 1950s contained class-1 MIs. Latest research has shown that a significant proportion of isolates of *Acinetobacter baumanii*, *Pseudomonas aeruginosa*, *E. coli*, and *Klebsiella pneumoniae* have class 1 integrons (Cambray et al., 2010).

**Table 3:** Class 2, 3, and 4 integron incidence and predominance in Gram-positive and Gram-negative microbial species (Deng et al., 2015).

Class 2 integrons			
Bacterial strain	Integron prevalence and the wide range of gene cassettes	Sampling	References
*Escherichia coli*	7.4% (31/417); *dfrA1-sat2-aadA1* (77.4%, 24/31), *estX-sat2-aadA1* (19.4%, 6/31) and *estX-sat2-△aadA1* (3.2%, 1/31)	BfT-GermVet monitoring study, Germany, 2004–2006	Kadlec and Schwarz (2008)
*Enterobacteriaceae*	34.9% (52/149); II2 (Tn7), III2 (*estX-sat2-aadA1-orfX*, most widely distributed) and IV2 (*aadA1*, first reported)	*E. coli* and *K. pneumoniae* strains from swine and chickens, Portugal	Machado et al. (2008)
*Escherichia coli*	One out of 322	Irrigation water and associated sediments, El Paso, Presidio and Weslaco	Roe et al. (2003)
*Escherichia coli*	3.0% (3/100)	Spain	Vinué et al. (2008)
*Escherichia coli*	3.6% (4/111); *dfrA1-sat1-aadA1*	The preliminary study, Guangzhou, China	Su et al. (2006)
*Coliforms*	2.7% (5/183)	Rivers in the northern region of Turkey	Ozgumus et al. (2009)
*Pseudomonas aeruginosa*	19.5% (23/118); *dfrA1-sat1-aadA1*, first report of class 2 integron in this species of bacteria	A preliminary study, Guangzhou, China	Xu et al. (2009)
*S. enteritidis*	4.3%; *estX-sat2-aadA1*	Poultry samples, Japan	Ahmed et al. (2005)
*S. enterica*	85 contemporary multidrug resistants D-Tartrate-Positive isolates; *dfrA1-sat1-aadA1*	*S. enterica* Serovar Paratyphi B isolates Germany, 1995–2001	Miko et al. (2003)
*S. sonneii*	93% (2/43)	Senegal adults diagnosed with diarrhea	Gassama-Sow et al. (2006)
*Shigella flexneri*	100% (58/58); *dfrA1-sat1-aadA1*	Samples of stool from people suffering from sporadic diarrhea, China, 2005–2006	Zhu et al. (2011)
*E. faecalis*	Two strains harboring class 1 and 2 integrons; *dfrA1-sat1-aadA1*, first evidence of class 2 integron in G+ bacteria	A preliminary study, Guangzhou, China	Xu et al. (2010)
Class 3 integrons			
*Escherichia coli*	*ges1/oxa10:aac* (6)	Switzerland	
*Serratia marcescens*	*imp1/aacA4*	Japan	Collis et al. (2002)
*Klebsiella pneumoniae*	*ges1/oxa10: aacA4*	Portugal’s critical care unit patient’s urine	Ploy et al. (2003)
Class 4 integrons			
*Vibrio cholerae*	Collection de I’Institut Pasteur (CIP)		Shibata et al. (2003) and Poirel et al. (2010)
*V. metschnikovii*			Shibata et al. (2003)

The clinical class 1 integron-integrase gene, intI1, is a good indicator of pollution because: (1) it is connected to genes that confer resistance to antibiotics, disinfectants, and heavy metals; (2) it is found in a wide range of pathogenic and nonpathogenic bacteria; (3) its abundance can change quickly because its host cells can have quick generation times; and (4) a single DNA sequence can measure the relative abundance of multiple bacteria (Gillings et al., 2015).

Integrons gene cassettes evolve antimicrobial resistance in primary clinical settings (Zhu et al., 2017; Ghaly et al., 2020b). These cassettes connected to integrons have been gathered from various genomes from different microbial environments. Despite claims to the contrary, it is believed that not all resistance genes found in microbial systems are represented by the resistance cassettes found in integrons and relatively encode resistance genes coding for nucleotidyl transferases, acetyltransferases, and β-lactamases (Partridge et al., 2018). Systematic screening of integron cassettes, which encode novel virulence, antimicrobial-resistance mechanisms, and pathogenicity, unravels the mechanism of invasion and colonization in human hosts (Bengtsson-Palme et al., 2018). For instance, Oliver et al. (2015) stated that integrons are essential in evolving antibiotic tolerance in *P. aeruginosa,* an opportunistic human pathogenic microbe.

Similarly, Class 4 integron was initially discovered in specific *Vibrio cholerae* chromosomes as a special form of integron. Additionally, it was a part of γ-proteobacterial genomes (Sabbagh et al., 2021). Class 4 integrons have also been a significant source of antibiotic tolerance and bacterial genome development (Poirel et al., 2018). Class 1 integrons were discovered in 1989 and predominate, especially in clinical contexts (Bush and Bradford, 2020; Ghaly et al., 2021a). Complete integrons are not self-moving components in and of themselves.

Furthermore, resistant integrons share many characteristics, including their ability to move, short cassette arrangements, and usually carrying only antibiotic-tolerance genes. On the other hand, these shared characteristics have not inherited traits of integron ancestors but rather the outcome of strong selection pressure during human antibiotic usage. Given the growing issue of antibiotic resistance, understand how bacteria evolve antibiotic resistance. The bulk of gene cassettes containing genes for antibiotic tolerance is acquired, reorganized, and expressed using mobile integrons (MIs), standard components (Qadri et al., 2021; Souque et al., 2021; Sheikh et al., 2021c). These elements, commonly coupled with transposons and conjugative plasmids, have aided pathogenic bacteria to evolve resistance.

Genetic platforms aid the molecular mechanism regarding the integron-mediated shuffling of antibiotic-tolerance genes. For instance, it has been demonstrated that constitutive amplification of the integrase enzyme speeds up the emergence of chloramphenicol tolerance by causing the deletion of cassettes between Pc and the resistance cassette and the development of co-integrates among copies of the integron (Barraud and Ploy, 2015). Molecular studies revealed that carbapenem resistance in *P. aeruginosa* is known to be a significant threat globally by the world health organization (WHO) and is set as an ‘extremely significant for scientists to develop novel antibiotic agents (San Millan et al., 2015). Specific reports suggest that integrons such as IntI1 catalysis of cassette insertion depend on specific factors (Vit et al., 2021). For instance, in *Vibrio cholerae*, the endogenous IntI was inactive in other bacterial hosts, proving the role of host factors needed for integron integrases (Vit et al., 2021).

### Integrons as genetic markers to provide an estimation of antimicrobial resistance dissemination

AMR is a serious health problem for humans and animals, and it is spread over various environmental environments. It is believed to be a continuing phenomenon that will pose severe threats to all dimensions of life, including animals, humans, and other organisms found environment. It is emerging as the prime focus of an investigation by scientific communities around the globe. The key player in antibiotic tolerance/resistance genes is integrons, which are promoter-less genetic factors carried from one pathogen to another (Cambray et al., 2010). To initiate a broad-spectrum approach, there is an urgent need to evolve methods to detect antibiotic resistance timely. Forefront to tackle these issues, a conjoint approach such as the “One Health approach” relies on integrated surveillance based on analysis of food, veterinary disease, public health, and the environment (Sikkema and Koopmans, 2016).

**Figure 5:**
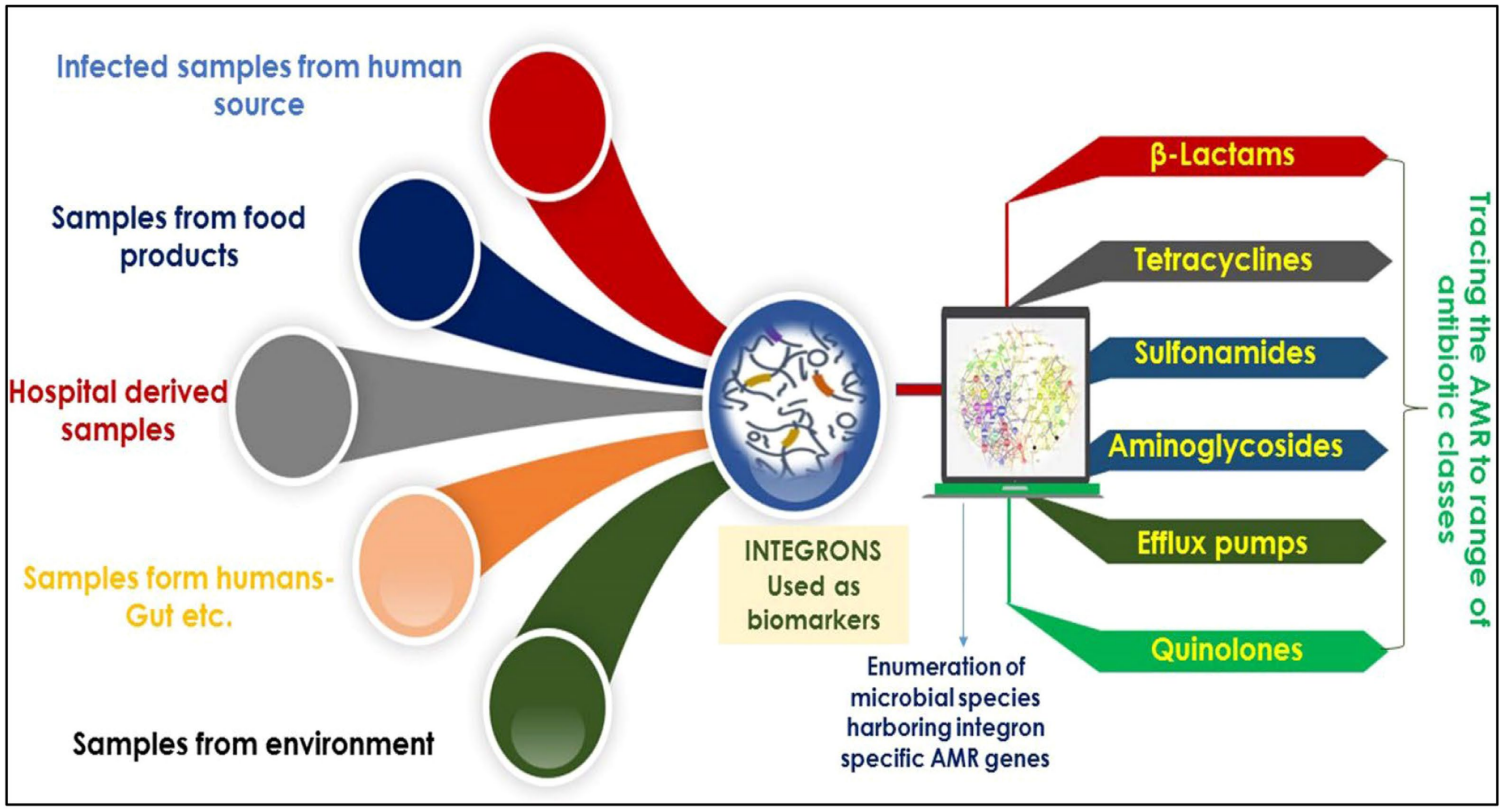
An overview showing the pivotal potential of integrons as biomarkers for the identification of AMR against a range of antibiotics/drugs.

Regarding recent studies, integrons are potentially employed as molecular markers to deliver an exact, reliable estimation of AMR dissemination across diverse habitats. Integrons are pivotal potential biomarkers currently employed to identify microbes harboring AMR against a range of antibiotics/drugs (Figure 5). For instance, analysis of integron-associated gene cassettes by targeted sequencing validated the role of integrons to identify the diversity of AMRs from complex microbial communities (Ghaly et al., 2020b). Reports suggest that integrons strongly provide a reliable and complete assessment of AMR expansion (Amos et al., 2015; Gillings et al., 2015). As mentioned in the introduction, integrons are of three main classes, viz., grossly involved in AMR spread. In particular, they are strongly linked to gram-negative bacteria (GNB) multidrug tolerance/resistance (Leverstein-van Hall et al., 2003; Barraud et al., 2014).

The class 1 integron is majorly found in humans and animals, whereas class 3 is found in samples collected from diverse environments (Tchuinte et al., 2016). Class 1 integrons mediate lateral transfer of DNA in-between pathogenic and commensal bacteria and thus accumulate antibiotic resistance genes in humans relying on the consumption of antibiotics. For example, in Betaproteobacteria based, class 1 integrons intersect the human food chains and can move between different locations among other bacterial species (Gillings et al., 2008). Moreover, Chainier et al. (2017) assessed the carriage of GNB in human and cattle samples by using the “One Health” approach based on cultivation-independent and cultivation-dependent approaches to check for the existence of integrons. They found class 1 integrons as expected, followed by class 2, with class 3 being more common in cattle than humans (Chainier et al., 2017). The study by Gianluca Corno et al., which examined a freshwater system of a lake–river–lake continuum, showed that class 1 integrons are a good proxy for anthropogenic pollution. They also suggest that this genetic platform is a significant driver of aminoglycoside resistance genes, including high-risk ARGs, a grave concern for human health (Corno et al., 2023).

Similarly, Barraud et al. recently reported the potential of integrons as predictive biomarkers for identifying antibiotic resistance in acute sepsis conditions (Barraud et al., 2022). In conjunction with previous reports, the study suggests integrons as a successful predictive marker for acquired antibiotic resistance through the direction in GNB-positive blood cultures (Barraud et al., 2014). Azizi et al. (2021) evaluated the identification and classification of integrons (class 1, 2, and 3) in strains of *Acinetobacter baumannii,* posing a severe problem for public health in Iran. The finding identified class 1 integrons are predictive biomarkers to validate the existence of MDR phenotypes in the clinical situation. Earlier, PCR-based amplification in Southern Taiwan identified class 1 integrons associated with resistance gene cassettes in *Pseudomonas aeruginosa* (Hsiao et al., 2014). De Paula et al. (2018) identified 40 resistance markers of clinical relevance to humans by subjecting the metagenomic samples to PCR amplification using markers specific to class 1, 2, and 3 integrons. They identified resistance markers against antibiotic agents like, sulfonamide, and quinolones.

## Conclusion and future perspectives

The serious global issue of AMR has put immense health-related pressure on humans and forced specialists to devise strategies to cope with resistance mechanisms evolved by microorganisms. Amid the heavy flow of resistance mechanisms from the environment to humans or vice versa, integrons have played a pivotal part in worsening the growth of tolerance through the expression and interchange of tolerance gene cassettes among different species of bacteria. The last decade has revealed the natural face of integrons and their development as the enquiring spectacle of curious clinical distress. These segments of microbial DNA are diverse in their existence and mechanisms and are reported to generate novel microbial genomes that trigger AMR dissemination and complexity. As explained, this immense health importance of integrons prompted us to provide a holistic view of AMRs with special reference to antibiotic resistance, its dissemination, and mobility through integrons. We propose that understanding the structure, functions, mechanism of AMR dissemination, and diversity of microbes identified through integrons will speed up our efforts to overcome AMR globally. As a result, successful mitigation techniques for AMR in humans and the environment are grossly limited due to a poor understanding of integron diversity, its dissemination mechanism, cross-mobility, and complexity. Moreover, we propose the following strategies to combat the microbial resistance mechanism:

- In-depth understanding of molecular structure and functions of integrons by in-silco analysis.
- In-depth investigation of integron evolution mechanism that drives the acquisition of AMR gene cassettes.
- Decrease the routes through which the resistant bacterial species contaminate humans.
- Reduce a load of antibiotics on the microbial pathogens to avoid selection pressure.
- Enumerate the realistic data on antibiotic resistance genes among major environmental habitats such as rivers, agricultural fields, hospitals, communities, etc.

Timely intervention accounting for mobile resistance gene evolution and mobility will prevent the devastating emergence of microbial resistance species threatening humans throughout the globe. We believe rising novel resistance genes in diverse microbial habitats through integrons would further hinder our efforts to devise treatment strategies, such as targeting integrons by antibiotics with adjuvants to inhibit SOS response. Development of new synthetic as well nature-based antimicrobials to combat the AMR (Chakravorty et al., 2020; Terreni et al., 2021; Bhat et al., 2022a,b; Mir et al., 2022a,b; Ashraf et al., 2023). At the end of 2020, there were 43 antibiotics in clinical development, of which, 15 were Phase I, 13 in Phase II, and 13 in Phase III to combat AMR. They include tetracycline derivatives (eravacycline), fourth generation fluoroquinolones (delafloxacin), new combinations between one β-lactam and one β-lactamase inhibitor (meropenem and vaborbactam), siderophore cephalosporins (cefiderocol), new aminoglycosides (plazomicin), and agents in development for treating drug resistant TB (pretomanid) (Terreni et al., 2021). Our review further bolsters the idea that successful therapeutic plans should focus on integrons, merging antibiotics with additives that restrict integrase function by suppressing the SOS response, favoring resistant bacterial species (Hocquet et al., 2012), limiting the selective pressure on the evolution of integrons. Furthermore, If we successfully manage integron and cassette creation, we may be able to create new biochemical routes for antimicrobial resistance employing integrons as a base to identify new enzymes. To effectively treat patients and employ antibiotics, it is helpful to be aware of the existence of integrons and gene cassettes.

## Author contributions

MM and BB designed the work. BB and RM wrote the manuscript and designed the figures. MM, BB, RM, HQ, and RD critically revised and edited the manuscript. All authors have read and approved the manuscript.

## Funding

The study was financially supported by Science and Engineering Research Board, Department of Science and Technology (SERB-DST) Govt. of India New Delhi; vide Project Grant No: TAR/001213/2018. We highly appreciate SERB-DST for financial assistance.

## Conflict of interest

The authors declare that the research was conducted in the absence of any commercial or financial relationships that could be construed as a potential conflict of interest.

## Correction note

A correction has been made to this article. Details can be found at: 10.3389/fmicb.2025.1681413.

## Publisher’s note

All claims expressed in this article are solely those of the authors and do not necessarily represent those of their affiliated organizations, or those of the publisher, the editors and the reviewers. Any product that may be evaluated in this article, or claim that may be made by its manufacturer, is not guaranteed or endorsed by the publisher.

## Footnotes

^1^http://integrall.bio.ua.pt/
