# Correction: Integrons in the development of antimicrobial resistance: critical review and perspectives

**DOI:** 10.3389/fmicb.2025.1681413

**Published:** 2025-09-12

**Authors:** Basharat Ahmad Bhat, Rakeeb Ahmad Mir, Hafsa Qadri, Rohan Dhiman, Abdullah Almilaibary, Mustfa Alkhanani, Manzoor Ahmad Mir

**Affiliations:** ^1^Department of Bio-Resources, School of Biological Sciences, University of Kashmir, Srinagar, India; ^2^Department of Biotechnology, School of Life Sciences, Central University of Kashmir, Ganderbal, India; ^3^Department of Life Sciences, National Institute of Technology (NIT), Rourkela, Odisha, India; ^4^Department of Family and Community Medicine, Faculty of Medicine, Al Baha University, Al Bahah, Saudi Arabia; ^5^Department of Biology, College of Science, Hafr Al Batin University of Hafr Al-Batin, Hafar Al Batin, Saudi Arabia

**Keywords:** antimicrobial resistance, antibiotic stewardship, horizontal gene transfer, integrons, pathogenicity

The sentence “An integron is generally defined by the presence of an integrase gene (*intI*) and a proximal primary recombination site (attI) (Figure 1; Partridge et al., 2009; Xu et al., 2011a).” was incorrect at publication. The sentence has been updated to “An integron is typically characterized by the presence of an integrase gene (intI) located near a primary recombination site known as attI (Deng et al., 2015).”

The caption of Figure 1 “Schematic representation of a class 1 integron. The basic integron platform consists of the following: intI, a gene for the integron integrase; Pc, an integron-carried promoter; attI, the integron-associated recombination site; and gene cassettes, sequentially inserted into an array via recombination between attI and the cassette associated-recombination sites, attC (Gillings, 2014; Ghaly et al., 2020a).” was incorrect at publication. The sentence has been updated to “A schematic diagram of a class 1 integron displays its essential components: the P1 promoter, which drives gene cassette transcription; the P2 promoter, typically inactive; the int gene encoding the integrase enzyme; the attI1 integration site; the qacEΔ1 gene conferring resistance to quaternary ammonium compounds; the sul1 gene for sulfonamide resistance; an orf5 of unknown function; and associated P promoters regulating expression of resistance genes. The *attC* site on gene cassettes is specifically recognized by the integrase for recombination (Deng et al., 2015).”

The paragraph “The *attC* domain consists of 2 simple sites, R″ and R′, L′ and L″, respectively, each made up of two conserved “core sites” (7 or 8 bp) (Bouvier et al., 2005). The RH consensus sequence has several connections to the RH simple site and includes the R′ and R″ sites. Similarly, the L′ and L″ parts of the LH consensus sequence are structurally and functionally similar to the LH. The integrase's ability to distinguish among the LH and RH sites in the attC could describe the position of integrated gene cassettes. Additionally, it looks as though L″ is more significant in terms of orientation (Bouvier et al., 2005). In addition to being necessary for orientation, the LH simple site promotes RH activity. (Partridge et al., 2009). A framework known as a gene cassette, typically not detectable during integrations but becomes an essential part of the integron once integrated, connects the *attC* sites to a single ORF in most cases (Deng et al., 2015).” in the **attC sites** section was incorrect at publication. The paragraph has been updated to “The *attC* region consists of two symmetrical sites, each comprising conserved short core sequences (7–8 base pairs) designated as R′ and R″, and L′ and L″. The R′ and R″ sequences align with the RH consensus, while L′ and L″ correspond to the LH consensus. These sequences are believed to guide the integrase in recognizing orientation-specific integration. The L″ site is especially important for determining insertion direction, and the LH site enhances the recombination efficiency of the RH site. Typically, an *attC* site is linked to a single open reading frame (ORF), forming a gene cassette. These cassettes, although sometimes independent, become a functional part of the integron upon integration (Deng et al., 2015).”

The paragraph “Gene cassettes are small movable components carrying a single gene, typically without a promoter or recombination site (*attC*). Gene cassettes are linear when integrated into the C1 integron but circular when left unintegrated or before site-specific insertion (Domingues et al., 2012). They can appear as a separate circular DNA molecule that cannot be maintained stable throughout cell division or as a linear DNA molecules formed when the free circular element is inserted into the integron in a particular orientation (Mazel, 2006). Prior research has shown that the structural nature of integrons usually lacks cassettes in the variable area (Deng et al., 2015).

Gene cassettes generally lack promoters, although having a coding sequence, which acts as the system's mobile component, and the majority of cassettes encode resistance to a wide variety of antibiotics, with over 130 different antibiotic resistance genes identified to date through distinctive *attC* sites (Hall and Collis, 1995). Most antibiotic families, such as -lactams, rifampicin, etc., and antiseptics from the quaternary ammonium compound family, are resistant to such cassettes (Hall and Collis, 1995).” in section **Gene cassettes** was incorrect at publication. The paragraph has been updated to “Studies have shown that the variable region of integrons may occasionally lack gene cassettes. When present, cassettes are integrated into the integron structure via site-specific recombination between *attI* and *attC* sites. These gene cassettes may exist as unstable circular DNA elements or as linear forms following directional insertion. Although they possess coding sequences, gene cassettes typically lack promoters and rely on the integrons promoter for expression. More than 130 distinct antibiotic resistance genes have been identified within these cassettes, conferring resistance to a broad range of antimicrobial agents, including β-lactams, aminoglycosides, chloramphenicol, streptothricin, trimethoprim, rifampin, erythromycin, quinolones, fosfomycin, lincomycin, and quaternary ammonium compounds.

Antimicrobial resistance arises through both rare spontaneous mutations and, more significantly, acquisition of resistance genes via vertical and horizontal gene transfer. The latter is often mediated by mobile genetic elements such as plasmids and transposons. Integrons, frequently located on these elements, facilitate rapid bacterial adaptation and are implicated in the rise of multidrug-resistant organisms, often referred to as “superbugs” (Deng et al., 2015).”

The original version of this article has been updated.

